# Normative uncertainties in public health foresight

**DOI:** 10.1093/eurpub/ckac129.122

**Published:** 2022-10-25

**Authors:** F Den Hertog, H Ruiter, H Hilderink

**Affiliations:** RIVM, Bilthoven, Netherlands

## Abstract

**Background:**

People value health differently, based on the norms and values. This is important when looking into the future trends in health and health care., and the perception of challenges they may arise from these trends.

**Methods:**

Together with stakeholders from a broad range of health-related professions, we identified several challenges for public health and healthy environments. Professionals working on these subjects came up with different goals and different ways to achieve them. These normative goals in health policies provide uncertainties in foresight, because they can be either synergetic or conflicting.

**Results:**

In the Dutch Public Health foresight study of 2014 four societal challenges for public health and healthcare were identified and formulated, as follows: (1) to keep people healthy as long as possible and cure illness promptly; (2) to support vulnerable people and enable social participation’ (3) to promote individual autonomy and freedom of choice; and (4) to keep healthcare affordable. In 2022 we will update and deepen these perspectives on public health and at the same time we will broaden the usability of working with this method by focussing on environmental health.

**Conclusions:**

To explicitly distinguish different perspectives on health leads to a better understanding and dealing with normative uncertainties by taking into account the trade-offs between competing interests and values. The challenge then is to identify policy options that have positive effect from different perspective, the win-wins.

